# Supplementary material for: AN OPTIMIZED METHOD TO DIFFERENTIATE HL60 CELLS INTO NEUTROPHIL-LIKE CELLS
Source: bioRxiv. 2026 Jan 7:2026.01.06.697988. Preprint. [Version 1] doi: 10.64898/2026.01.06.697988 (PMC12803222; doi:10.64898/2026.01.06.697988)
Supplement: Supplement 3 [file NIHPP2026.01.06.697988v1-supplement-3.pdf]

bioRxiv preprint doi: https://doi.org/10.64898/2026.01.06.697988; this version posted January 8, 2026. The copyright holder for this preprint (which was not certified by peer review) is the author/funder, who has granted bioRxiv a license to display the preprint in perpetuity. It is made available under aCC-BY 4.0 International license.

SUPPLEMENTARY INFORMATION

Supplementary Figure 1
Chemotaxis parameters for individual DMSO and A1D4 dHL60 cells

A-I. Dot plot graphs showing chemotaxis parameters after 60 min of migration towards fMLF (**a-c**), LTB_4 (**d-f**) N=3, IL-8 (**g-i**) N=3. For each chemoattractant the following parameters are displayed: average cell speed (**a, d, g**), which represents cells' average speed during migration from each independent experiment; Euclidean distance travelled (**e, i, m**), which shows cells' distance traveled in a straight line; and directionality (**f, j, n**), which represents the ratio of Euclidean distance to total accumulated distance traveled. Black bar represents mean ± interquartile range.

Supplementary Figure 2
Nuclear protein expression in dHL60 cells

A. Western blot showing levels of LMNA, LMNC, LBR and GAPDH in cell lysates from DMSO and A1D4 dHL60 cells. Molecular weights (kDa) are indicated on the left. N=6.

B-D. Bar graphs showing expression levels via band intensity of LBR (**b**), LMNA (**c**), and LMNC (**d**) normalized to GAPDH expression. *P* values calculated using two-tailed t test. Data are presented as mean ± s.e.m.

E-F. Representative microscopy images of multiple fixed dHL60 cells stimulated with 100nM LTB_4 for 15 min, immunostained with LBR (yellow) and LMNA/C (magenta) in **e** and FLAP (green) in **f**, both co-stained with DAPI (cyan). Scale bar is 20μm.

Supplementary Video 1
Representative widefield microscopy movies of dHL60 cells migrating for 60 min toward fMLF. Nuclei are visualized by a purple circle and cell tracks are shown as a trailing, colored line. The associated differentiation condition and time stamp are provided at the top left and right of each movie panel. Scale bar is 200μm. Representative of three independent experiments.

Supplementary Video 2
Representative widefield microscopy movies of dHL60 cells migrating for 60 min towards fMLF, LTB_4, or IL-8. Nuclei are visualized by a purple circle and cell tracks are shown as a trailing, colored line. The associated differentiation condition is provided on the left of the two rows of movies. The chemoattractant and time stamp are provided in the bottom and top right of each movie panel. Scale bar is 200μm. Representative of three independent experiments.

bioRxiv preprint doi: https://doi.org/10.64898/2026.01.06.697988; this version posted January 8, 2026. The copyright holder for this preprint (which was not certified by peer review) is the author/funder, who has granted bioRxiv a license to display the preprint in perpetuity. It is made available under aCC-BY 4.0 International license.

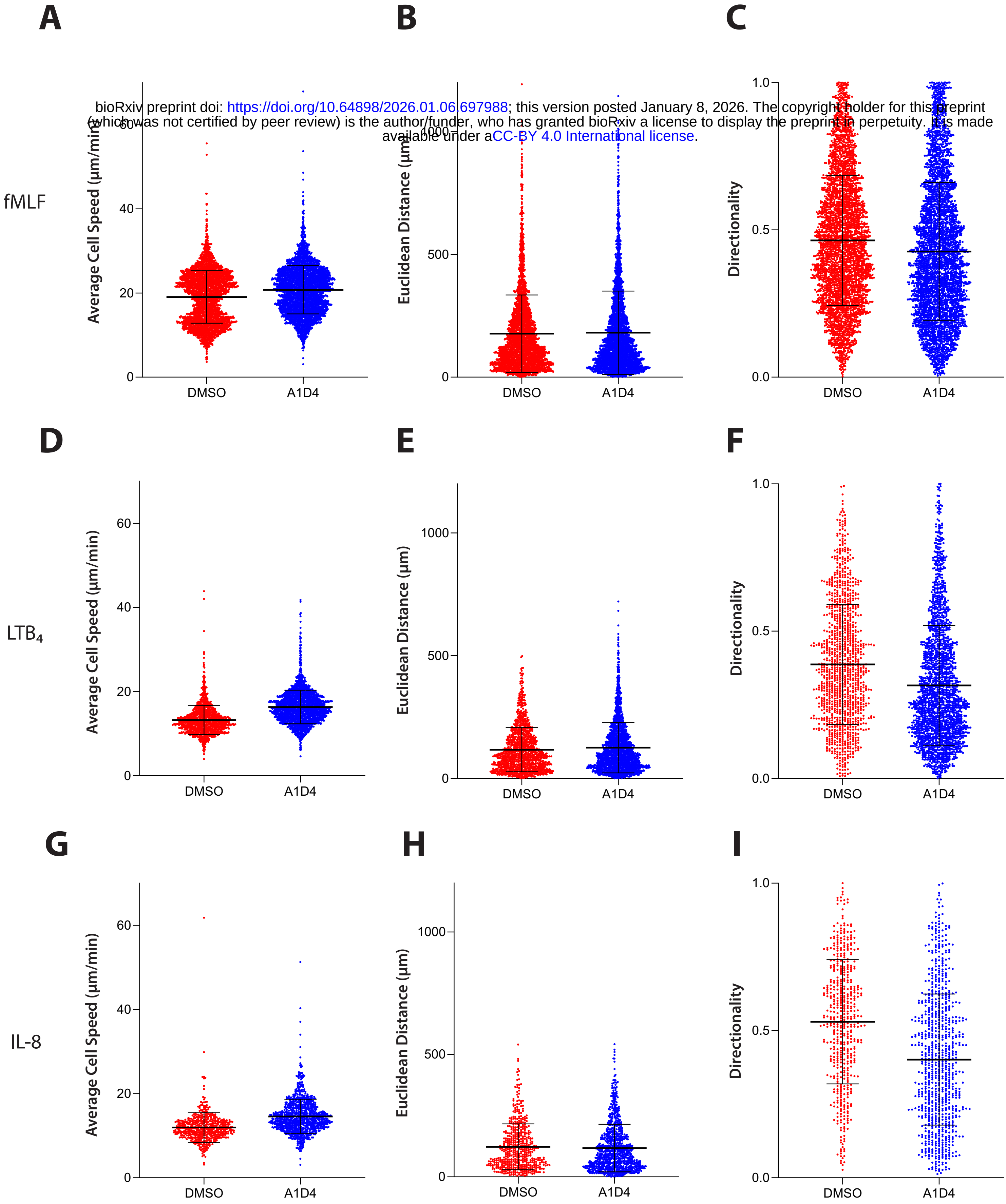

bioRxiv preprint doi: https://doi.org/10.64898/2026.01.06.697988; this version posted January 8, 2026. The copyright holder for this preprint (which was not certified by peer review) is the author/funder, who has granted bioRxiv a license to display the preprint in perpetuity. It is made available under aCC-BY 4.0 International license.

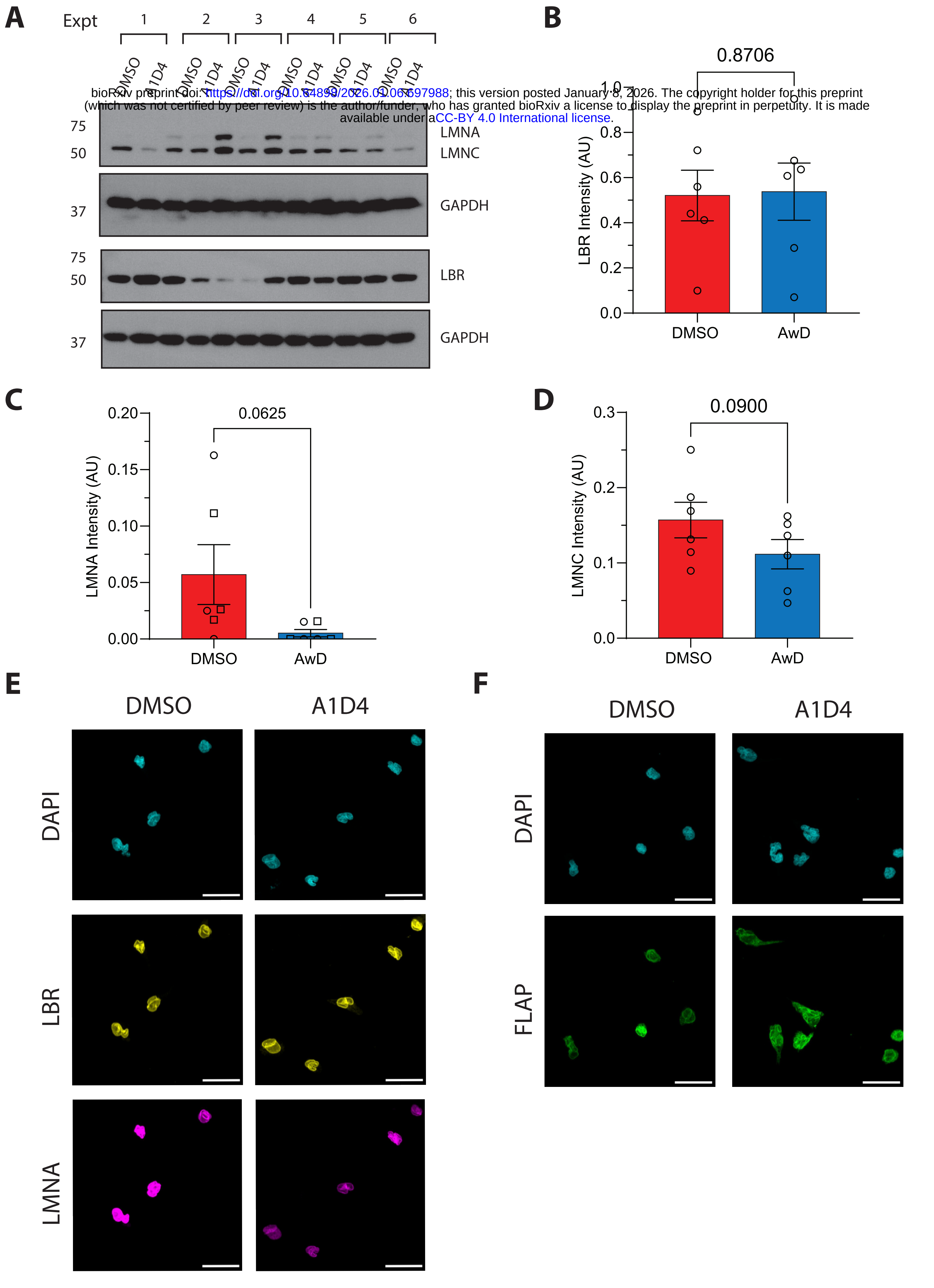

Collie et al.

Supplemental Figure 2
